# Range‐wide population genetic structure of the Caribbean marine angiosperm *Thalassia testudinum*

**DOI:** 10.1002/ece3.4443

**Published:** 2018-08-29

**Authors:** Kor‐jent van Dijk, Eric Bricker, Brigitta I. van Tussenbroek, Michelle Waycott

**Affiliations:** ^1^ School of Biological Sciences Environment Institute Australian Centre for Evolutionary Biology and Biodiversity The University of Adelaide Adelaide South Australia Australia; ^2^ Unidad Académica Puerto Morelos Instituto de Ciencias del Mar y Limnología Universidad Nacional Autónoma de México (UNAM) Cancún México; ^3^ Department of Environmental Sciences The University of Virginia Charlottesville Virginia; ^4^ Department for Environment and Water State Herbarium of South Australia Botanic Gardens and State Herbarium Adelaide South Australia Australia

**Keywords:** gene flow, genetic differentiation, Gulf of Mexico, long‐distance dispersal, seagrass, turtle grass

## Abstract

Many marine species have widespread geographic ranges derived from their evolutionary and ecological history particularly their modes of dispersal. Seagrass (marine angiosperm) species have ranges that are unusually widespread, which is not unexpected following recent reviews of reproductive strategies demonstrating the potential for long‐distance dispersal combined with longevity through clonality. An exemplar of these dual biological features is turtle grass (*Thalassia testudinum*) which is an ecologically important species throughout the tropical Atlantic region. Turtle grass has been documented to have long‐distance dispersal via floating fruits and also extreme clonality and longevity. We hypothesize that across its range, *Thalassia testudinum* will have very limited regional population structure due to these characteristics and under typical models of population structure would expect to detect high levels of genetic connectivity. There are very few studies of range‐wide genetic connectivity documented for seagrasses or other sessile marine species. This study presents a population genetic dataset that represents a geographic area exceeding 14,000 km^2^. Population genetic diversity was evaluated from 32 *Thalassia testudinum* populations sampled across the Caribbean and Gulf of Mexico. Genotypes were based on nine microsatellites, and haplotypes were based on chloroplast DNA sequences. Very limited phylogeographic signal from cpDNA reduced the potential comparative analyses possible. Multiple analytical clustering approaches on population genetic data revealed two significant genetic partitions: (a) the Caribbean and (b) the Gulf of Mexico. Genetic diversity was high (*H*_E_ = 0.641), and isolation by distance was significant; gene flow and migration estimates across the entire range were however modest, we suggest that the frequency of successful recruitment across the range is uncommon. *Thalassia testudinum* maintains genetic diversity across its entire distribution range. The genetic split may be explained by genetic drift during recolonization from refugia following relatively recent reduction in available habitat such as the last glacial maxima.

## 1 INTRODUCTION

Dispersal of propagules is critical for population maintenance in sessile marine organisms as it is essentially the only mechanism for such species to move other than the gametes themselves. Dispersal can most simply be thought of as the movement of an organism from a source (parent) to a settlement site (Cowen & Sponaugle, 2009; Kinlan & Gaines, 2003; Kinlan, Gaines, & Lester, 2005; Weersing & Toonen, 2009). Benthic marine organisms generally release propagules into the water column which subsequently are transported by water movements (Shanks, Grantham, & Carr, 2003); however, this migration can only be considered successful when the progeny is deposited in a suitable environment (Siegel, Kinlan, Gaylord, & Gaines, 2003) and become a reproductive individual (Kinlan & Gaines, 2003). It is important to note that the dispersal range of aquatic plants is generally larger than terrestrial species (Les, Crawford, Kimball, Moody, & Landolt, 2003; Santamaría, 2002), meaning direct dispersal can occur over a range of meters to hundreds of kilometers (McMahon et al., 2014).

Quantifying long‐distance dispersal (LDD) in a systematic and meaningful way is complex, particularly in marine environments. Effective dispersal distances in the marine environment may be extremely large and usually do not reflect the mean displacements of the propagules (Kinlan et al., 2005). Event‐based migrations often determine colonization to new locations and are important for maintaining genetic connectivity (Kinlan & Gaines, 2003; Les et al., 2003; Ouborg, Piquot, & Van Groenendael, 1999). In such situations, LDD can only be assessed retrospectively with the use of genetic markers. A typical approach to estimate LDD is to assess the relative frequencies of alleles at marker loci in populations and statistically test or model the likelihood of the allele frequencies being observed. The indirect approach to measuring/modeling connectivity based on genetic data is widely used but is dependent on many assumptions that are rarely met (i.e., short generation time, no overlapping generations, etc.). Nevertheless, the outcomes give valuable insight into the relationships of populations.

Previous reviews have attempted to describe the relationship between genetic differentiation and marine connectivity using various genetic markers (Kinlan & Gaines, 2003; Selkoe et al., 2016; Weersing & Toonen, 2009). These works demonstrate a limited correlation between genetic differentiation of populations (*F*_ST_) and dispersal distance. The discrepancy these studies describe is partially attributed to the difficulty in comparing *F*_ST_ results when utilizing different types of genetic markers and the scale that these are used at. Additionally, a debate has been taking place on the appropriate application of the various measures of genetic differentiation (Hedrick, 2005; Heller & Siegismund, 2009; Jost, 2008; Meirmans & Hedrick, 2011), which has highlighted some concerns on the use of microsatellites for population genetics. Marine species possessing wide geographic ranges and long dispersal potential allow for valuable case studies to examine the spatial scale of genetic connectivity.

This study investigates the benthic seagrass, *Thalassia testudinum* Banks ex König (turtle grass), which has a wide geographic range residing in shallow tropical and subtropical waters across the Western Atlantic Ocean. Here, it plays an essential role as a foundation species forming extensive meadows and providing numerous ecosystem services (Green & Short, 2003; Van Tussenbroek et al., 2006). *Thalassia testudinum* occurs predominately in the Caribbean tropical marine biogeographic province. This region lost physical connectivity with the Tethys Sea following the closure of the Isthmus of Panama approximately 3 Ma (O'Dea et al., 2016), a process which initiated much earlier (7 Ma; Muss, Robertson, Stepien, Wirtz, & Bowen, 2001). Many phylogeographic studies exist for this region, particularly on fish and corals, but few have covered the total range of their study species (e.g., Chaves‐Fonnegra, Feldheim, Secord, & Lopez, 2015; Purcell, Cowen, Hughes, & Williams, 2009). In such large‐scale studies, estimates of connectivity (measured as gene flow) have shown inconsistencies in the genetic structure of the target taxa. Some studies report high gene flow between distant sites (e.g., Purcell et al., 2009) while others have shown to be significantly structured and partitioned (e.g., Chaves‐Fonnegra et al., 2015). Although ubiquitous throughout its range, populations of *T. testudinum* are disconnected by deeper water areas, unsuitable substratum for the establishment, and major river discharges (Van Tussenbroek et al., 2006). *Thalassia testudinum* has floating propagules (buoyant fruits), which allow for LDD beyond the range of local populations (van Dijk, van Tussenbroek, Jiménez Durán, Márquez Guzmán, & Ouborg, 2009). Like all perennial seagrasses, it is capable of longevity through clonality, a function of vegetative growth through rhizome extension and the repetitive formation of new potentially independently functional plant units or ramets (van Dijk & van Tussenbroek, 2010). The combination of LDD, clonality, and longevity produces a highly complex demographic structure.

In this study, we evaluate genetic diversity, connectivity, structure, and phylogeographic relationships among populations of *T. testudinum*. This species is an exemplary case study on range‐wide connectivity in this area as this species is one of the most common submarine plants found in the region and will represent the first investigation where population structure of a tropical seagrass species is described across its entire range.

We propose that the current distribution of *T. testudinum* is dependent on genetic connectivity across large spatial scales. We hypothesize that *T. testudinum* displays the capacity to remain highly connected, as evident by weak geographic structure. It is likely *T. testudinum* maintains connectivity through moderate to high levels of gene flow that we expect to occur across most of the Western Atlantic despite the large geographic region this species inhabits. The combination of long‐distance dispersal with longevity through clonality enables the extended spatial extent of connected populations even with relatively constrained recruitment processes. We will also examine if there is evidence of recent historical barriers, such as sea‐level changes associated with the most recent glacial maxima (~26 Kya, e.g., Peltier & Fairbanks, 2006) or deeper divergences such as those associated with the closure of the Panamanian Isthmus (~2.8 Mya O'Dea et al., 2016) have influenced population divergences. Both time periods altered oceanic conditions and habitat availability along with the direction and distribution of currents influencing the potential population size and genetic diversity of *T. testudinum*. For contemporary analysis, we use species‐specific microsatellite markers to assess genetic structure and estimate gene flow. Historical contractions or expansions will be investigated through intraspecific phylogenetic relationships with one nuclear and three chloroplast DNA sequence loci.

## 2 MATERIALS AND METHODS

### 2.1 Ethics statement

Sample permits were issued by SAGARPA for Mexico, and Ministerio del Ambiente for Costa Rica; specific permission was not required for the other (CARICOMP) sampling sites. Other collections were made under the auspices of permits to The University of Virginia.

### 2.2 Sampling locations

**Figure 1:**
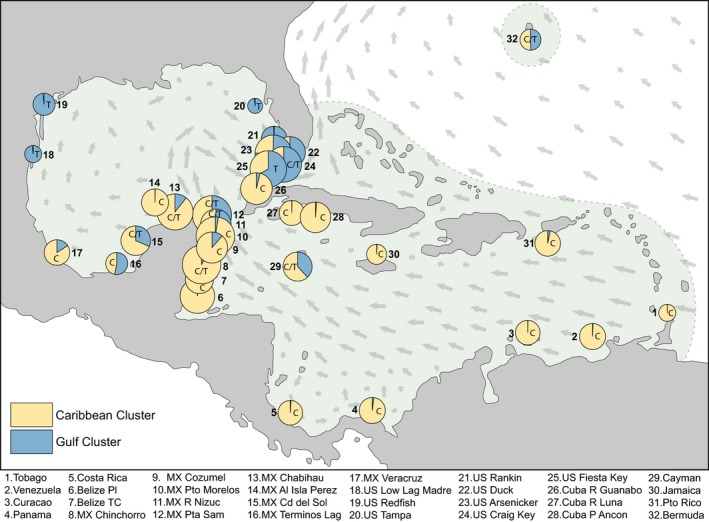
Populations of *Thalassia testudinum* and phylogeographic assignment. Collection sites of 32 *T. testudinum* populations in its total distribution range (edge delimited by solid line). Population details are found in Supporting Information Table S1 in Appendix S2. Each pie depicts the relative assignment proportions of each population to clusters 1 and 2 (Caribbean and Gulf of Mexico). The diameter of each pie corresponds to the population's relative allelic diversity (Table 1). Within each pie, the attribution to rbcLa haplotype C or T is also shown. The gray arrows show the directions and intensities of the major superficial currents (Gyory, Mariano, & Ryan, 2005)

Thirty‐two populations of *T. testudinum* were collected throughout the species range (Figure 1); fifteen of these were sampled by collaborators of CARICOMP (The Caribbean Coastal Marine Productivity Program) and other institutions (Supporting Information Table S5 in Appendix S2). For each population, two groups of 15 samples (foliar shoots, 2 m apart) were collected, and the two groups were 500–2,000 m apart (depending on local conditions). The Belize–Placencia population was collected by grab‐sampling at irregularly spaced intervals at least 5 m apart. Populations from US‐Rankin, US‐Duck, and US‐Arsenicker were obtained by randomly collecting 30 samples at a similar spatial scale from genotypic data used in Bricker, Waycott, Calladine, and Zieman (2011). Pairwise geographic distances between the populations ranged from 4 to 4,249 km (Supporting Information Table S7 in Appendix S2). Distances were measured as the shortest possible distance by sea. Procedures for sample collection, DNA extraction, and amplification followed van Dijk et al. (2009) using TTMS‐GA6, TTMS‐GA8, TTMS‐GA12, TTMS‐TCT58, TTMS‐GGT59, TTMS‐GA72, TTMS‐GT77, TTMS‐GT104, and Th1MS microsatellite loci for genotyping (van Dijk, Waycott, van Tussenbroek, & Ouborg, 2007). PCR products were analyzed by capillary electrophoresis on a MegaBACE 1000 DNA Sequencing System (GE Healthcare, Amersham Biosciences, Little Chalfont, United Kingdom). Alleles were scored from chromatographic traces using Genetic Profiler Suite.

Clonality within populations was determined with GenClone (Arnaud‐Haond & Belkhir, 2007), including indices for clonal richness (*R*) and probabilities for obtaining identical genotypes *p*_gen_ and *p*_sex_ (Arnaud‐Haond, Duarte, Alberto, & Serrão, 2007). Only one single copy of each multilocus genotype (MLG) was used in the following analyses. Genalex (Peakall & Smouse, 2006) was used to calculate the allele frequency, observed heterozygosity (*H*_O_), and unbiased expected heterozygosity ($H_{E}^{\prime }$) with their corresponding standard deviations (Peakall & Smouse, 2006). The web version of Genepop (Raymond & Rousset, 1995) was used to estimate the inbreeding coefficient (*F*_IS_) for each population, also the significance of the global heterozygote excess and deficit was determined using the default parameters. The average number of alleles per locus (*A*) and the allelic richness (*A*_[n]_) for all populations were estimated with Hp‐Rare (Kalinowski, 2005) applying a rarefaction to 15 samples (Kalinowski, 2002).

### 2.3 Genetic structuring

To determine whether *T. testudinum* is subdivided into groups of shared ancestry across its range, a Bayesian assignment test was performed across all populations using Structure (Pritchard, Stephens, & Donnelly, 2000), assuming admixture and correlated allele frequencies. For each *K* (1 to 32), 20 independent runs (10^5^ iterations burn‐in and 10^7^ main) were performed. Summary statistics, ∆*K* as described by Evanno, Regnaut, and Goudet (2005), and graphs of the runs were generated with the web package CLUMPAK (Kopelman, Mayzel, Jakobsson, Rosenberg, & Mayrose, 2015). An analysis of molecular variance (ANOVA) was used to quantify how genetic diversity was distributed among individuals, populations, and clusters. Calculations were made with GenoDive (Meirmans & Van Tierden, 2004) using an infinite allele model (999 permutations). $F_{\mathrm{ST}}^{\prime }$ (Meirmans, 2006) was also estimated to correct for dependence of *F*_ST_ to the genetic variation of the used markers. A primary component analysis (PCA) was performed on the data as an independent approach to detect genetic patterns. The R package adegenet (Jombart, 2008) was used to do the calculations and depict the data.

### 2.4 Connectivity

Three estimators for pairwise genetic differentiation were calculated, *F*_ST_ (estimated as Θ Weir and Cockerham (1984)), $F_{\mathrm{ST}}^{\prime }$ (Hedrick, 2005), and *D* (Jost, 2008) using GenoDive (Meirmans & Van Tierden, 2004). To test for isolation by distance (IBD, Wright, 1943), a Mantel test was performed with each of these pairwise estimates with the web‐based program IBDWS where all three measures were correlated with the pairwise geographical distances of the 32 populations. The correlations and slopes of the relationships were tested by a reduced major axis (RMA) regression applying 1,000 randomizations. IBD testing was determined assuming two‐dimensional dispersal, applying a geographic log‐transformation (Rousset, 1997). IBD was also calculated at a regional scale assuming two genetic clusters.

The relative number of migrants per generation was calculated with the divMigrate function in the R package diveRsity (Keenan, McGinnity, Cross, Crozier, & Prodöhl, 2013) to illustrate connectedness between populations. The *G*_ST_ and newly developed $\hat{\mathrm{Nm}}$ (Alcala, Goudet, & Vuilleumier, 2014) statistics were used to estimate the relative values of gene flow. Connectivity was visualized with the qgraph package in R. Estimates of contemporary migration rates were conducted in BayesAss (Wilson & Rannala, 2003); due to the limited number of populations, the program could handle a modified version of the software was used (courtesy of Bruce Rannala). Default settings were used with a burn‐in of 1.0 × 10^6^ iterations and a 4.0 × 10^7^ run. Network analysis following the approach of Dyer and Nason (2004) was conducted in GENETIC STUDIO b.131 (Dyer, 2009). The population topology was developed using allelic data without a priori assumptions on any particular structure such as the data being organized by geographic region. The overall arrangement of the network is determined by the genetic relationships among populations where the edge lengths depict the among population component of genetic variation representing connectivity. The nodes represent the populations where the sizes reflect the populations’ genetic variability (Dyer & Nason, 2004).

### 2.5 Phylogenetic analysis

One sample per site was selected to perform an initial test on amplification and sequencing success, and this was done for the nuclear ribosomal intergenic spacers ITS‐1 and ITS‐2 (primers ITS 1,2,3 and 4, White, Bruns, Lee, & Taylor, 1990). The chloroplast coding region rbcLa (Kress et al., 2009) and noncoding intergenic spacers, trnH‐psbA (Kress, Wurdack, Zimmer, Weigt, & Janzen, 2005), trnL‐F spacer (Taberlet, Gielly, Pautou, & Bouvet, 1991), atpF‐atpH, and psbK‐psbI (Kim Ki‐Joong unpublished) were also tested. Routine PCR protocols were performed in 35 μl reactions using MyTaqHS (Bioline, London, UK) and 1 μl of diluted template (1:5). The PCR cycling conditions were [95°C 2 m (95°C 20 s, 58°C 20 s, 72°C 30 s) 35 cycles, 72°C 2 m], and products were sent for sequencing to BGI (Beijing Genomics Institute, Shenzhen, Guangdong, China). Only one locus was used for an expanded study; rbcLa and 4–5 MLGs were amplified for each population. The sequences were processed and aligned with the software Geneious (Biomatters, New Zealand).

## 3 RESULTS

### 3.1 Genetic diversity

A total of 996 seagrass ramets were genotyped and after removal of redundant MGLs, 662 individual genotypes remained for analysis. These had a total of 137 alleles, with loci having between 8 and 23 alleles. The probability of obtaining the same genotype by a sexual event estimated by *p*_sex_ was generally low. Marginal populations with very low allelic diversity, like Bermuda and Laguna Madre, had high probabilities. Clonality varied among populations with clonal richness ranging from *R *=* *0.21 up to *R *=* *1.00, with an average of *R*_all_ = 0.66 (Table 1). Analysis of the MLGs revealed that clone mates were only detected within populations and not among. Gene diversity was generally high with *H*_E_ ranges between 0.35 and 0.75. Two sites, MX‐Pta Sam and US‐Arsenicker, showed heterozygosity deficits. The rarefied allelic richness (*A*_[15]_) ranged between 3.2 and 6.9 (Table 1).

**Table 1:** Summary population clonal and genetic measures estimated for population samples of *Thalassia testudinum* across its range

Pop #	Pop.	*N*	*G*	*R*	*p*_gen_ max	*p*_sex_ max	*A*	*A*_[15]_	*H*_O_	*H*_E_	*SD*	*F*_IS_	P H_deficit_	P H_excess_
1	Tobago	30	16	0.52	9.84 × 10^−05^	2.95 × 10^−03^	3.4	3.4	0.542	0.550	0.046	0.016	0.133	0.867
2	Venezuela	30	21	0.69	5.52 × 10^−05^	7.52 × 10^−04^	5.2	4.8	0.671	0.597	0.056	−0.126	0.993	0.008
3	Curacao	30	18	0.59	3.17 × 10^−06^	9.50 × 10^−05^	4.9	4.7	0.728	0.688	0.045	−0.061	0.964	0.036
4	Panama	30	13	0.41	4.16 × 10^−08^	1.25 × 10^−06^	5.2	—	0.667	0.703	0.074	0.054	0.046	0.954
5	Costa Rica	29	16	0.54	8.60 × 10^−07^	2.49 × 10^−05^	4.9	4.8	0.694	0.688	0.032	−0.010	0.322	0.678
6	Belize Pl	34	23	0.67	2.82 × 10^−08^	9.58 × 10^−07^	6.9	6.2	0.676	0.719	0.027	0.060	0.007	0.994
7	Belize TC	30	10	0.31	2.56 × 10^−06^	7.67 × 10^−05^	5.6	—	0.789	0.727	0.032	−0.090	0.316	0.684
8	MX Chinchorro	36	24	0.66	1.11 × 10^−09^	4.01 × 10^−05^	7.7	6.9	0.782	0.768	0.029	−0.019	0.894	0.106
9	MX Cozumel	33	33	1.00	1.26 × 10^−08^	—	6.2	5.5	0.687	0.721	0.028	0.048	0.048	0.952
10	MX Pto Morelos	35	31	0.88	6.10 × 10^−09^	7.22 × 10^−11^	7.7	6.6	0.681	0.715	0.051	0.049	0.015	0.985
11	MX R Nizuc	23	22	0.95	1.66 × 10^−07^	—	6.1	5.8	0.747	0.721	0.039	−0.038	0.008	0.992
12	MX Pta Sam	36	36	1.00	1.46 × 10^−08^	—	7.8	6.4	0.676	0.718	0.040	0.060	**0.001**	0.999
13	MX Chabihau	36	21	0.57	1.40 × 10^−08^	5.05 × 10^−07^	7.2	6.8	0.778	0.746	0.041	−0.044	0.515	0.485
14	MX Al Isla Perez	36	27	0.74	1.39 × 10^−07^	1.71 × 10^−06^	5.4	4.8	0.687	0.653	0.049	−0.054	0.968	0.032
15	MX Cd del Sol	36	17	0.46	2.19 × 10^−08^	7.67 × 10^−07^	5.9	5.8	0.680	0.727	0.032	0.067	0.022	0.978
16	Mx Terminos Lag	36	25	0.69	2.60 × 10^−05^	9.94 × 10^−06^	4.4	4.2	0.627	0.631	0.044	0.008	0.039	0.961
17	MX Veracruz	36	20	0.54	6.95 × 10^−08^	2.50 × 10^−06^	5.1	4.8	0.611	0.670	0.030	0.090	0.254	0.746
18	US L Lag Madre	30	20	0.66	6.79 × 10^−03^	1.85 × 10^−01^	3.4	3.2	0.350	0.347	0.102	−0.009	0.070	0.930
19	US Redfish	30	23	0.76	3.31 × 10^−04^	8.33 × 10^−04^	4.3	4.0	0.435	0.449	0.097	0.032	0.376	0.624
20	US Tampa	30	9	0.28	2.18 × 10^−04^	3.21 × 10^−02^	3.0	—	0.506	0.440	0.104	−0.161	0.947	0.054
21	US Rankin	26	24	0.92	1.73 × 10^−05^	2.68 × 10^−04^	5.1	4.6	0.532	0.560	0.070	0.050	0.013	0.987
22	US Duck	27	27	1.00	2.71 × 10^−06^	—	6.2	5.5	0.580	0.602	0.064	0.037	0.008	0.993
23	US Arsenicker	30	28	0.93	1.25 × 10^−06^	5.23 × 10^−09^	7.3	6.2	0.639	0.682	0.065	0.064	**0.001**	0.999
24	US Craig Key	29	24	0.82	7.24 × 10^−08^	1.38 × 10^−06^	7.1	6.4	0.741	0.725	0.038	−0.022	0.427	0.573
25	US Fiesta Key	30	22	0.72	3.14 × 10^−08^	8.89 × 10^−08^	7.3	6.6	0.732	0.729	0.042	−0.005	0.063	0.938
26	Cuba R Guanabo	30	28	0.93	1.62 × 10^−07^	2.48 × 10^−07^	6.3	5.7	0.722	0.724	0.033	0.002	0.095	0.905
27	Cuba R Luna	30	14	0.45	8.59 × 10^−07^	2.58 × 10^−05^	5.0	—	0.730	0.690	0.033	−0.060	0.936	0.064
28	Cuba P Ancon	30	15	0.48	1.68 × 10^−05^	5.05 × 10^−04^	6.1	6.1	0.756	0.673	0.038	−0.127	0.953	0.047
29	Cayman	30	16	0.52	6.17 × 10^−07^	1.85 × 10^−05^	5.8	5.7	0.743	0.694	0.052	−0.074	0.948	0.052
30	Jamaica	29	12	0.39	2.35 × 10^−07^	6.81 × 10^−06^	5.0	—	0.713	0.746	0.021	0.046	0.429	0.571
31	Pto Rico	30	20	0.66	5.30 × 10^−06^	1.59 × 10^−04^	5.1	4.9	0.656	0.640	0.037	−0.024	0.924	0.076
32	Bermuda	29	7	0.21	6.61 × 10^−03^	1.75 × 10^−01^	4.2	—	0.619	0.647	0.085	0.047	0.180	0.820

*Notes*

*N*: number of genotyped ramets; *G*: number of genets; *R*: clonal richness (*G*−1/*N*−1); *p*_gen_ max: highest probability a clonal identity per population; *p*_sex_ max: highest probability for obtaining an identical genotype through sex; *A*: average number of alleles per locus; *A*_[15]_: average allelic richness (rarefaction to 15 samples, populations marked with—did not reach number of samples required); *H*_O_: observed heterozygosity; *H*_E_: unbiased expected heterozygosity with its standard deviation (*SD*); *F*_IS_: inbreeding coefficient.

Global levels of heterozygote excess or deficit plus significance levels (bold values are significantly out of Hardy–Weinberg equilibrium) after applying a Bonferroni correction.

### 3.2 Genetic structuring

Structure analysis and plotting ∆*K* describe maximum genetic structure at two regional clusters, although subdividing the populations into three (*K* = 3) also resulted in above average structure, but less markedly (Supporting Information Figures S1, S2, and Table S3 in Appendix S1). The two major clusters can roughly be subdivided into the Caribbean (cluster 1) and the Gulf of Mexico (cluster 2), with some populations having intermediate membership. The PCA ordination arranged populations in concordance to Structure models (Supporting Information Figure S4 in Appendix S1). Again, Caribbean and Gulf of Mexico populations were grouped separately with some intermediate populations. The population graph generated with Genetic Studio depicts a similar distribution of genetic diversity (Supporting Information Figure S5 in Appendix S1).

Hierarchical partitioning of variance among populations and clusters (Supporting Information Table S2 in Appendix S2) indicates that the assignments are based on slight shifts in the allelic frequencies and not on strong isolation of the regions. The variance within each cluster (*F*_SC_ = 0.160) was higher than that between clusters (*F*_CT_ 0.084). Due to the nature of this analysis, assignment to clusters was determined by highest proportion of membership. The global measure of genetic structuring is *F*_ST_ = 0.161 (calculated with no partitioning among populations). Genetic differentiation measures among the sampled populations increased considerably when the variance was standardized to its potential maximum (Meirmans, 2006), resulting in $F_{\mathrm{ST}}^{\prime }$ = 0.531 over the whole study area, with $F_{\mathrm{CT}}^{\prime }$ = 0.370 among regions and $F_{\mathrm{SC}}^{\prime }$ = 0.478 among populations within regions.

### 3.3 Phylogeography

In order to evaluate and assess diversity and applicability for phylogeographic analysis, six loci were tested across a subset of samples, which consisted of samples distributed throughout the range of this study. Loci ITS1, trnL‐F, and psbK‐I failed to produce usable sequences and were excluded. Loci ITS2 and trnH‐psbA sequenced well, and atpF‐H sequenced well through the first 160 bp, but none had variable base positions (Supporting Information Table S15 in Appendix S2). rbcLa had one variable site, and this locus was used for the broader study to infer phylogeography. Only two haplotypes were found across the range, and several populations had both haplotypes. Haplotype distribution followed a similar pattern as the genotypic data for *K* = 2 (Figure 1).

### 3.4 Connectivity

**Figure 2:**
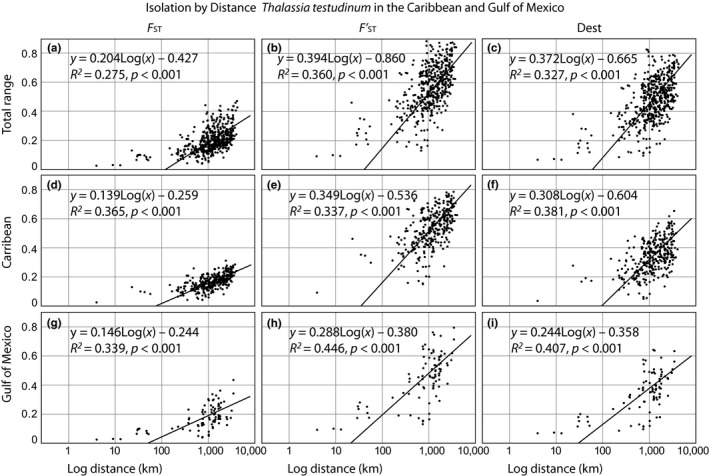
Isolation by distance for *Thalassia testudinum*. Isolation by distance (IBD) was calculated for populations of *Thalassia testudinum* through the whole range (Graphs a–c**)**. Based on genetic assignments with Structure, IBD was also calculated within each cluster (Caribbean (d–f) and Gulf of Mexico (g–i)). Populations sharing assignments to both clusters were included in both datasets if assignment proportions were between 0.3 and 0.7 (Supporting Information Table S3 in Appendix S1). Correlations were based on pairwise genetic distance as *F*_ST_ (Weir & Cockerham, 1984), pairwise standardized $F_{\mathrm{ST}}^{\prime }$ (Hedrick, 2005; Jost, 2008), and Jost's *D*_EST_ (Jost, 2008) against the pairwise geographic distance (determined by de shortest distance over water possible; see Supporting Information Tables S3–S11 in Appendix S2). Log‐geographic corrections were applied to all analyses according to Rousset (1997)

Measures of genetic differentiation varied greatly among populations (Supporting Information Tables S3–S11 in Appendix S2). The fixation index *F*_ST_ calculated the smallest range of values (0.025–0.470), whereas *F’*_ST_ is always higher (0.090–0.900). Values of Jost's *D* fall generally between the two previous measures ranging from 0.068 to 0.789 (Figure 2). Much of the differentiation variance occurs between populations that are more than 1,000 km apart, particularly for *D* (0.119–0.789, Figure 2c), indicating that some very distant population are similar in allelic composition, while others are almost totally differentiated. Significant IBD is observed across the sampled region with all analytical measures. Independent IBD graphs of both the Caribbean and Gulf of Mexico clusters show similar patterns of connectivity (Figure 2d–i).

**Figure 3:**
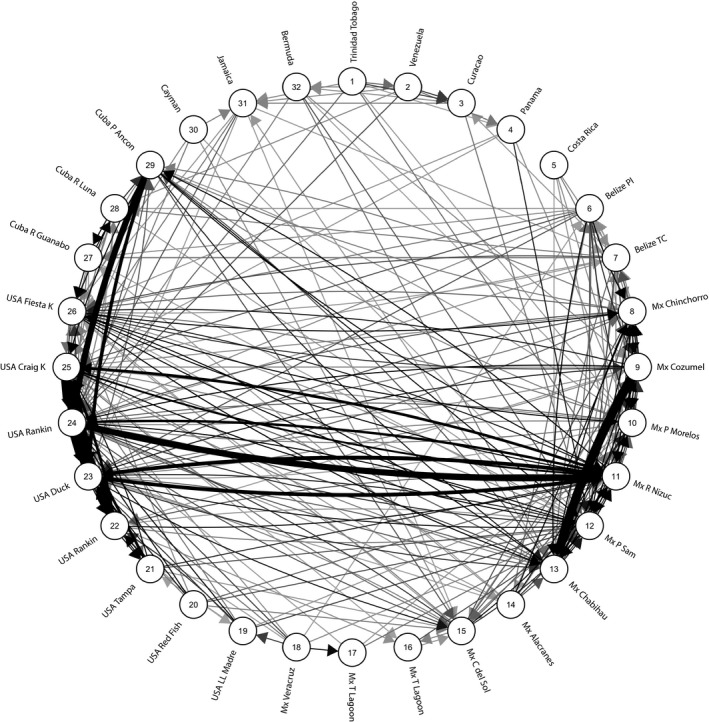
Connectivity graph of 32 populations of *Thalassia testudinum* in the Caribbean and the Gulf of Mexico. Relative migration between populations pairs for all 32 populations was calculated and plotted with the R package diveRsity (Keenan et al., 2013) using the divMigrate function (Sundqvist, Zackrisson, & Kleinhans, 2013) and using $\hat{\mathrm{Nm}}$ (Alcala et al., 2014) as the connectivity (migration) estimate (Supporting Information Table S13 in Appendix S2). The divMigrate function plots the relative asymmetric migration between populations, from microsatellite allele frequency data. A lower threshold of relative migration of 0.14 was used to eliminate uninformative edges, and edges were scaled by width and color saturation when above 0.40. Wider and darker edges represent the most connected sites of this study with the highest relative connectivity (1.0) between 23.US Craig Key and 24.US Arsenicker. The numbers within the nodes represent the populations as in Table 1

Genetic connectivity calculated as the relative number of migrants between population pairs indicate that nearby populations are more interconnected than distant ones, with some significant migration between the Yucatan Peninsula and the Florida Keys (Figure 3 based on $\hat{\mathrm{Nm}}$ Figure 3 and *G*_ST_ Supporting Information Figure S6 in Appendix S1, Tables S13 and S14 in Appendix S2). A Bayesian approach to infer migration into the sampled populations in the most recent generations (BayesAss analysis) shows that successful settlement of new or second‐generation individuals is usually below 1% and never surpasses 2% of sampled MLGs (Supporting Information Table S12 in Appendix S2). Comparing the outcomes from five different measures of connectivity and population distinctiveness, as relative migration based on $\hat{\mathrm{Nm}}$ (Figure 3) and *G*_ST_ (Supporting Information Figure S6 in Appendix S1), BayesAss (Supporting Information Table S12 in Appendix S2), network analysis (Supporting Information Figure S5 in Appendix S1) and PCA (Supporting Information Figure S4 in Appendix S1) demonstrates that the broad scale processes dominate the data whichever method of analysis utilized.

## 4 DISCUSSION

In this study, we investigated the hypothesis that the biological traits of *Thalassia testudinum* provide for genetic connectivity over large distances leading to low levels of genetic structure across the extant range. Genotypic multilocus data and sequence data show that genetic structure is low and that long‐term integrated gene flow occurs across large to very large distances. We also found that genetic diversity was very high compared to similar studies (see Section 4.1). Genetic diversity is highest near Cuba and tapers off toward the periphery of the species distribution. This indicates that the current pattern of genetic diversity can be explained by the central–marginal hypothesis (Eckert, Samis, & Lougheed, 2008). The analyzed populations are divided into two regions corresponding with the major basins, the Caribbean and the Gulf of Mexico (Figure 1), with indications of admixed ancestry of populations at the boundary and northern periphery. The genetic differentiation between the two regions only accounts for 8.4% of the overall genetic variation for all samples. Long‐distance dispersal of floating fruits likely maintains connectivity among populations (van Dijk et al., 2009). Migration leading to recruitment is rare on an “annual” basis, but it remains a recurrent possibility within the long lifespan of the genets of *T. testudinum*, which is estimated to be decades to centuries (Arnaud‐Haond et al., 2012; van Dijk & van Tussenbroek, 2010). These results corroborate our hypothesis of high connectivity and low genetic structure. The distribution of haplotypes confirms the phylogeographic distribution into two major regions (Figure 1) and indicates this split is not an ancient one.

### 4.1 Genetic diversity throughout the range

Measures of genetic diversity (*H*_E_, *A*, Table 1) show that *T. testudinum* is genetically diverse throughout the tested range, with a trend of decreased expected heterozygosity (*H*_E_) and alleles per locus (*A*) toward the periphery of the species biogeographic range (Bermuda, Tobago, US Low Laguna Madre and Tampa). This trend is most likely driven by reduced gene flow toward the edges, an increased likelihood of population extinction and recolonization, leading to genetic drift (Eckert et al., 2008). All range boundaries, but the most northern populations, are determined by substrate availability or water clarity. The northern populations (Florida east coast and Bermuda) are under higher environmental pressures (e.g., low light and temperature) as physiological conditions are suboptimal. All the mentioned conditions can result in a smaller effective population size and consequently, a higher probability of inbreeding and genetic drift (Eckert et al., 2008; Vucetich & Waite, 2003). Historical climate‐driven fluctuations might also have contributed to this distribution and need to be analyzed further. Various of the peripheral populations are fixed (have only one allele) for one or more loci (US‐Low Laguna Madre; two loci, US‐Red Fish; one locus, US‐Tampa; one locus and Bermuda; 1 locus).

The average genetic diversity expressed in *H*_E_ over all sites was 0.66, which is higher than results for whole range studies on other species of seagrass such as *Zostera marina* with *H*_E_ = 0.45 (Olsen et al., 2004), *Z. noltii* with *H*_E_ = 0.45 (Coyer et al., 2004), *Cymodocea nodosa* with *H*_E_ = 0.48 (Alberto et al., 2008), and *Posidonia oceanica* with *H*_E_ = 0.40 (Arnaud‐Haond, Migliaccio, et al., 2007).

Clonal growth, a key life‐history strategy for *T. testudinum,* was dominant in many of the populations (Table 1); Bermuda being most notable with clones detected at ≥750 m distance. Such large genets might be very old, with estimated ages of 1050‐1950y according to rhizome elongation rates (van Dijk & van Tussenbroek, 2010). Bermuda was the most isolated population of this study and has low genetic diversity (*H*_E_, *A*) and high clonality. Here, local genetic diversity was negatively affected by the restricted gene flow as propagules (either clonal or sexual) must cross ~1,300 km of open ocean before reaching suitable habitat. The open ocean barrier between Bermuda and other *T. testudinum* populations is a strong indicator of the dispersal potential of this species, a characteristic that is likely widespread across all the seagrass groups (Kendrick et al., 2012; McMahon et al., 2014; Waycott, Procaccini, Les, & Reusch, 2006).

Based on genetic and genotypic diversity, Laguna Madre populations appear to have established recently, with both sites having low allelic diversity and a high number of unique MLGs. This semiclosed coastal ecosystem has limited access to the main basin of the Gulf of Mexico, which lowers the probability of migrants entering the area (Quammen & Onuf, 1993). The most plausible explanations for this demography may be the relatively recent establishment of a few plants followed by high sexual reproduction, recombining the few alleles present. While inferential, this model of expansion is supported by the rapid expansion of *T. testudinum* in the lower Laguna Madre from 1965 to 1988, when this seagrass was only found in the most southern part of the system near passes, but then became widespread throughout the lower Laguna Madre (Kaldy & Dunton, 1999; Quammen & Onuf, 1993). This phenomenon of rapid establishment and local inbreeding has been identified in another species of seagrass, *Halodule wrightii,* in the upper‐ and lower Laguna Madre (e.g., Larkin, Maloney, Rubiano‐Rincon, & Barrett, 2017).

### 4.2 Connectivity

Gene flow was the most important microevolutionary force leading to homogeneity in genetic and allelic diversity among populations (Hedrick, 1999). *Thalassia testudinum* has an elevated capacity for sexually derived long‐distance dispersal (van Dijk et al., 2009; Kaldy & Dunton, 1999), and recent research indicates that vegetative dispersal in seagrasses could also be important (e.g., Bricker, Calladine, Virnstein, & Waycott, 2018; Smulders, Vonk, Engel, & Christianen, 2017). Thus, it is to be expected that neighboring populations are more related to each other than more distant ones (Isolation By Distance, Wright, 1943), even when these distant populations are hundreds of kilometers apart. This is confirmed by the pattern of IBD found in this study, which was consistent across all utilized measures of genetic differentiation (*F*_ST_, $F_{\mathrm{ST}}^{\prime }$, or *D*_EST_)_._

When the populations of the Gulf of Mexico and the Caribbean basin are analyzed as separate entities, IBD relationships are very similar. The potential habitat in the Gulf of Mexico is not continuous but is intermitted along the northeast shores. We therefore expected these sites to be less connected, although this is not reflected in the outcomes. Interestingly, many of the Gulf sites (Laguna Madre and Tampa Bay) contained populations with the highest differentiation values to other sites. It is also important to note that geographic distance between populations is the shortest distance over water possible, which will not be the typical path of dispersal of propagules as local surface and subsurface currents are rarely linear (Lems‐de Jong, 2017) and the shortest distance will therefore be an underestimate. Hydrodynamic modeling would be necessary to produce more accurate estimates (e.g., White et al., 2010) as environmental and climatological factors can impact the route of gene flow (van Dijk et al., 2009).

These data also speak to the ongoing mathematical controversies related to *G*_ST_‐based measures (Hedrick, 2005; Jost, 2008; Meirmans & Hedrick, 2011). For example, estimating gene flow with *F*_ST_ involves violating many assumptions of the Wright‐Fisher model for allelic inheritance (Whithlock & McCauley, 1999). Highly clonal plants are problematic for most Hardy–Weinberg‐based estimates of relationships among alleles as clonal‐based longevity results in many generations that might overlap by decades or centuries.

When we compare the differentiation measures in this study, *F*_ST_ yielded lower values than the other two measures, particularly at the larger geographic scales. When standardized, $F_{\mathrm{ST}}^{\prime }$ reached almost complete differentiation for some population pairs, highlighting the underperformance of *F*_ST_ when using highly polymorphic markers. Jost's *D* appears to best describe the geographic pattern in an IBD context; however, more studies are needed to evaluate its ecological significance as it measures different aspects of population structure (Jost, 2009). Leng and Zhang (2011) recommend the use of both *F*_ST_ and *D* to obtain a more comprehensive description of the microevolutionary processes that influence population differentiation. Most pairs of populations had limited gene flow (indicated by *F*_ST_ and *D *>* *0.15), but low *F*_ST_ and *D* values (<0.1) are also observed at distances above a 1,000 km, suggesting that long‐distance dispersal might occur, although this is not supported by the chloroplast sequence data. Relative migration based on the alternative measure of Nm (number of migrants per generation) that considers both *G*_ST_ and *D* in its estimate (formula (12) Alcala et al., 2014) demonstrates similar patterns of connectivity (Figure 3). Bayesian models of migration inferred through BayesAss (Table S12 in Appendix S2) confirm that dispersal has not occurred in recent generations.

### 4.3 Genetic structure

The genetic structuring of *T. testudinum* based on the distribution of genetic diversity is moderate (*F*_ST_ = 0.161) which is consistent with previous findings for *T. testudinum* in Mexico (van Dijk et al., 2009). And when standardized, this measure increases to $F_{\mathrm{ST}}^{\prime }$ = 0.531. To date, few studies have adopted this measure; therefore, genuine comparisons are not possible. The *F*_SC_ results for each of the two regions (Supporting Information Table S2 in Appendix S2) demonstrate that the genetic variance is very similar among biogeographic regions. The AMOVA results also show that there is no apparent gene flow barrier between the Caribbean and the Gulf of Mexico. Analogous studies on seagrasses at a similar scale using microsatellites found *F*_ST_ values that were twice as high as ours, as in the sister species *T. hemprichii* (*F*_ST_ = 0.353, Hernawan et al., 2017) and other genera (e.g., Arnaud‐Haond, Migliaccio, et al., 2007; Olsen et al., 2004).

Based on Bayesian modeling of allelic partitioning implemented in Structure, the most likely subdivision of all data was into two genetic clusters (or regions, i.e., *K* = 2). Ordination‐based analysis visualized as a PCA of the two most informative axes confirms the two‐region split (Supporting Information Figure S4 in Appendix S1). Phylogeographic sequence data support this outcome. This distribution can be possibly explained by the rapid radiation from two isolated refugia (possibly similar to what is occurring in the Laguna Madre) after one of the recent glacial maxima.

For all approaches, most of the Caribbean sites were fully assigned to the “Caribbean” cluster. The northern three sites of the Gulf of Mexico and some of the Florida Key populations fully assigned to the cluster “Gulf of Mexico.” Intermediate genetic assignments (sharing provenance/haplotypes from both clusters) are found along the boundary of the Gulf and Caribbean (Figure 1). In particular, populations on the western side of the Yucatan Peninsula have a strong influence from the Caribbean, possibly explained by a localized stepping‐stone type coastal dispersal along the continuous meadows of *T. testudinum* around the Yucatan Peninsula coast (van Dijk et al., 2009). The strong influence of the Caribbean cluster at Isla Perez and around Florida Bay and some populations at Florida Keys in the Gulf of Mexico can be explained by the directions of major ocean currents. The Caribbean Current develops into the Loop Current and flows northwards into the Gulf of Mexico until bending south along the northern coasts of the Gulf (arrows Figure 1), eventually joining the Florida Current. Bermuda is geographically independent of both regions and remarkably has a ~50%–50% association with the two genetic clusters. The presence of *T. testudinum* in Bermuda is an important example of how long‐distance dispersal does occur (even at the lowest sea levels, Bermuda was very distant) and that the fast‐flowing currents that lead into the Gulf Current can carry viable propagules, 1,300 km away from the nearest population. Fast‐flowing currents are a key environmental factor for *T. testudinum's* sexual dispersal as the fruits are buoyant. These fruits eventually open and the seeds, which are not buoyant, drop to the sea floor (Van Tussenbroek et al., 2016). The importance of asexual propagules is unknown and needs to be investigated. We expected this to be of significant importance, but identifying the occurrence is almost impossible. Hydrodynamic superficial dispersal modeling would help conceptualize the bulk flow of *T. testudinum* propagules and possibly explain how the species maintains such high genetic connectivity. It could also help elucidate how migration between the Caribbean and the Gulf of Mexico occurs.

## 5 CONCLUSION

The findings presented in this study deliver significant insight into the genetic diversity, clonality, connectivity, and overall range‐wide population structure of a important tropical seagrass species. Our results indicate that the marine angiosperm *Thalassia testudinum* has been able to persist and thrive across the wide range of marine environments of the Western Atlantic through clonality providing a stable base to release propagules which disperse widely, although appear to recruit at long distances infrequently. Genetic structure modeling and analyses of phylogeography using sequence data indicate that there is population structure at the scale of two major regions, the Caribbean and Gulf of Mexico. As expected, isolated populations such as Bermuda and Tampa Bay show higher levels of genetic differentiation. Lower Laguna Madre populations are genetically secluded from the rest of the Western Atlantic populations and appear to be extending their local range via sexual recruitment. Overall genetic differentiation between populations increases with distance and at the extremes of the range populations are almost completely isolated.

The extant populations of this keystone species exhibit high levels of genetic diversity with an indicative higher level of diversity is around western Cuba, an area where both lineages exchange genes more frequently. Although across the range as a whole, the scale of connectivity is large, the frequency of successful recruitment appears to be only moderate. In addition, although genetically diverse, the slow rate at which microevolutionary processes occur in this long‐lived clonal species does pose a risk under rapid environmental change scenarios such as climate change.

We provide evidence that demonstrates long‐distance migration is widespread and a variable process that brings new recruits (sexual and/or clonal) into local populations. Recent migration patterns (the last couple of generations, which could be hundreds to thousands of years) indicate more than 90% of recruits come from local sources. The fact that only two clusters are found with both genotypic (relatively fast‐evolving) and phylogeographic (relatively slow evolving) analysis tools indicates two separate recolonization events occurred in the Caribbean and Gulf of Mexico, which we propose aligns with recolonization following the last glacial maximum when range expansion was likely to be rapid and widespread for *Thalassia testudinum*.

## CONFLICT OF INTEREST

The authors have no conflict of interests.

## AUTHOR CONTRIBUTIONS

K.J.v.D is responsible for design, sample collection, data gathering, and analysis and led writing. E.B. is responsible for Florida Bay population collecting and data analysis and editing of the manuscript. B.I.v.T. involved in main supervision of PhD and is responsible for conception of ideas and interpretation of results and editing of the manuscript. M.W. is the main contributor to analytical approach, interpretation of results, and writing of manuscript. All authors approved the manuscript draft.

## DATA ACCESSIBILITY

DNA sequences: GenBank accessions See Supporting Information Table S15 in Appendix S2. Sampling locations: Supporting Information Table S1 in Appendix S2. Microsatellite genotypes: Dryad doi: https://doi.org/10.5061/dryad.g663p2b.
