# Supplementary material for: Range‐wide population genetic structure of the Caribbean marine angiosperm Thalassia testudinum
Source: Ecol Evol. 2018 Aug 29;8(18):9478–90. doi: 10.1002/ece3.4443 (PMC6194253; doi:10.1002/ece3.4443)
Supplement: Supplementary file 1 [file ECE3-8-9478-s001.docx]

*Ecology and Evolution*

**SUPPORTING INFORMATION**

**Appendix 1**

**Title: Range wide population genetic structure of the Caribbean marine angiosperm *Thalassia testudinum***

Kor-jent van Dijk

Eric Bricker

Brigitta I. van Tussenbroek

Michelle Waycott

**POPULATION STRUCTURE STATISTICS**

**
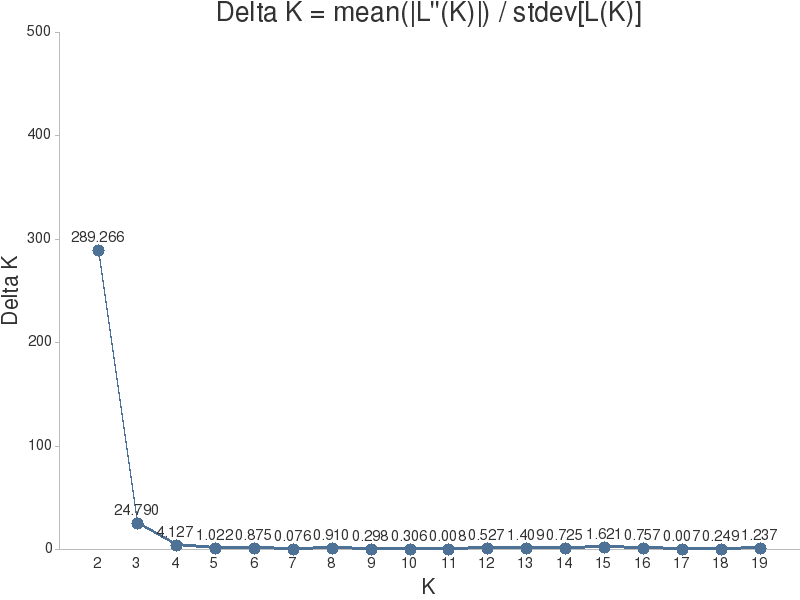
Figure S1** Graph representing the ∆K for *Thalassia testudinum*.

Thirty-two *Thalassia testudinum* populations in the Greater Caribbean and Gulf of Mexico where assessed using ∆K with the software package CLUPMAK (Kopelman *et al.*, 2015). Inferences were made for K=2 to K=31 as described by Evanno *et al.* (2005). Due to the nature of this *ad hoc* procedure ∆K for k=1is not possible to calculate, values for K=1 to K19 are only shown.


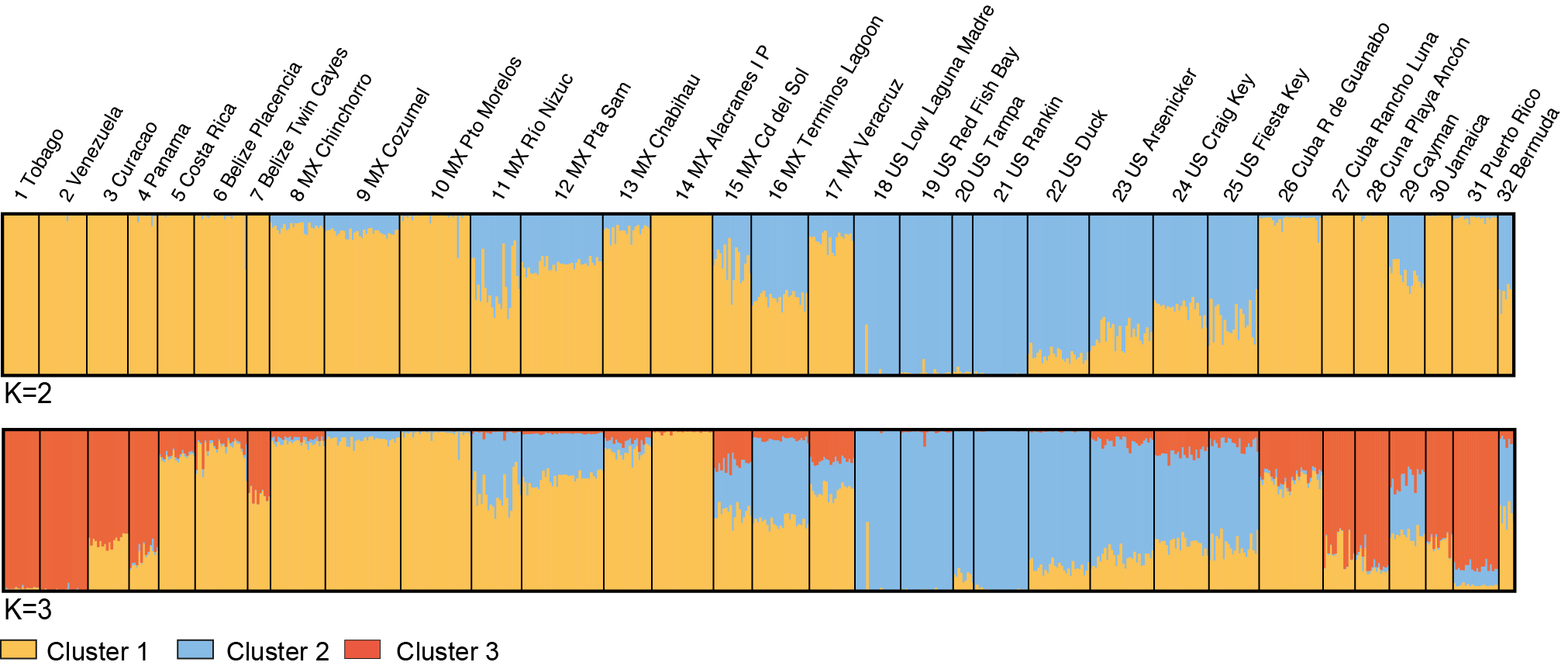


**Figure S2** Structure results *Thalassia testudinum*.

Graphical representation of the population sample’s coefficient of membership assuming 2 clusters (K=2, used for map) and for 3 clusters (K=3) inferred with STRUCTURE (Pritchard & Wen, 2003). Graphical representation is a summary graph of the major clusters generated in CLUMPAK (Kopelman *et al.*, 2015). K=2 and K=3 were plotted as these had elevated ∆K values.

**Table S3 Cluster membership values *Thalassia testudinum*.**

The proportion of membership of each population to one of the two or three inferred clusters (K=2 and K=3) calculated with STRUCTURE (Pritchard & Wen, 2003). The selected K values were based on the ∆K statistic (Evanno *et al.*, 2005), employing 10 independent runs for K=1 to K=32. Bayesian assignment procedure was performed with an admixture and correlated allele frequency model. Numbers in bold indicate probability of membership of more that 70%. Proportions of membership were determined in CLUMPAK (Kopelman *et al.*, 2015) by averaging the outcomes of the major clusters. K=2 and K=3 are presented as these had elevated ∆K values.

| **Population** | | **K=2** | | **K=3** | | |
| --- | --- | --- | --- | --- | --- | --- |
|  |  | **1** | **2** | **1** | **2** | **3** |
| 1 | Tobago | **0.998** | 0.002 | 0.016 | 0.006 | **0.978** |
| 2 | Venezuela | **0.993** | 0.007 | 0.007 | 0.007 | **0.986** |
| 3 | Curacao | **0.998** | 0.002 | 0.320 | 0.003 | 0.677 |
| 4 | Panama | **0.980** | 0.020 | 0.200 | 0.026 | **0.774** |
| 5 | Costa Rica | **0.991** | 0.009 | **0.831** | 0.023 | 0.146 |
| 6 | Belize Pl | **0.970** | 0.030 | **0.873** | 0.031 | 0.095 |
| 7 | Belize TC | **0.995** | 0.005 | 0.615 | 0.007 | 0.379 |
| 8 | MX Chinchorro | **0.918** | 0.082 | **0.911** | 0.032 | 0.057 |
| 9 | MX Cozumel | **0.885** | 0.115 | **0.936** | 0.062 | 0.002 |
| 10 | MX Pto Morelos | **0.975** | 0.025 | **0.971** | 0.023 | 0.006 |
| 11 | MX R Nizuc | 0.556 | 0.444 | 0.596 | 0.386 | 0.019 |
| 12 | MX Pta Sam | 0.686 | 0.314 | **0.700** | 0.282 | 0.018 |
| 13 | MX Chabihau | **0.893** | 0.107 | **0.858** | 0.073 | 0.069 |
| 14 | MX Al Isla Perez | **0.997** | 0.003 | **0.984** | 0.003 | 0.013 |
| 15 | MX Cd del Sol | 0.686 | 0.314 | 0.483 | 0.306 | 0.212 |
| 16 | Mx Terminos Lag | 0.470 | 0.530 | 0.432 | 0.512 | 0.056 |
| 17 | MX Veracruz | **0.841** | 0.159 | 0.635 | 0.193 | 0.171 |
| 18 | US Low Lag Madre | 0.020 | **0.980** | 0.024 | **0.972** | 0.003 |
| 19 | US Redfish | 0.015 | **0.985** | 0.007 | **0.979** | 0.014 |
| 20 | US Tampa | 0.019 | **0.981** | 0.096 | **0.899** | 0.005 |
| 21 | US Rankin | 0.007 | **0.993** | 0.009 | **0.987** | 0.005 |
| 22 | US Duck | 0.123 | **0.877** | 0.152 | **0.844** | 0.004 |
| 23 | US Arsenicker | 0.248 | **0.752** | 0.206 | **0.722** | 0.072 |
| 24 | US Craig Key | 0.414 | 0.586 | 0.294 | 0.562 | 0.144 |
| 25 | US Fiesta Key | 0.328 | 0.672 | 0.276 | 0.641 | 0.083 |
| 26 | Cuba R Guanabo | **0.963** | 0.037 | 0.678 | 0.037 | 0.285 |
| 27 | Cuba R Luna | **0.997** | 0.003 | 0.254 | 0.009 | **0.736** |
| 28 | Cuba P Ancon | **0.989** | 0.011 | 0.151 | 0.040 | **0.809** |
| 29 | Cayman | 0.619 | 0.381 | 0.333 | 0.359 | 0.308 |
| 30 | Jamaica | **0.992** | 0.008 | 0.306 | 0.014 | 0.679 |
| 31 | Pto Rico | **0.968** | 0.032 | 0.034 | 0.117 | **0.849** |
| 32 | Bermuda | 0.505 | 0.495 | 0.476 | 0.459 | 0.065 |


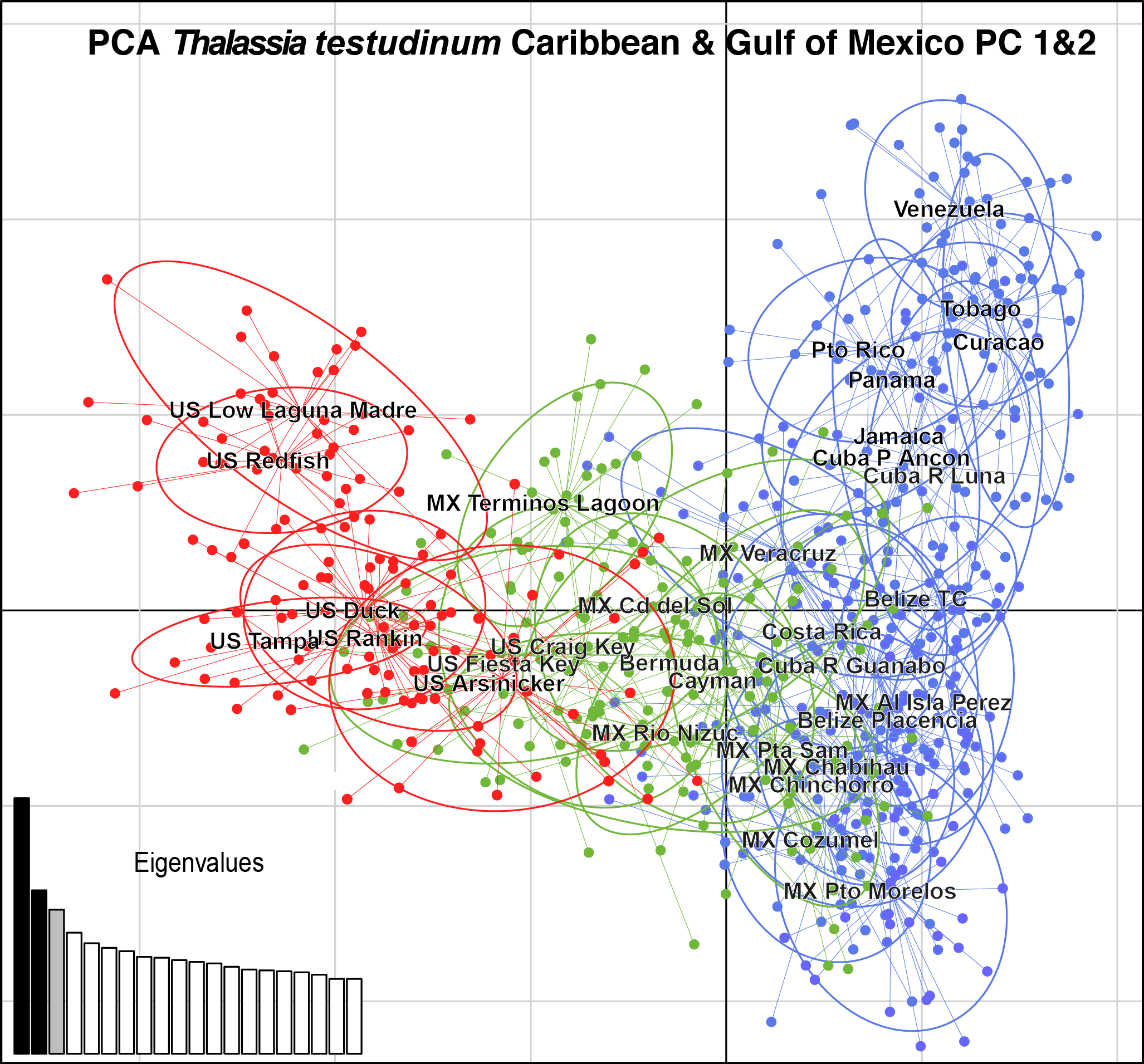


**Figure S4** Principal Component Analysis for 32 populations of *Thalassia testudinum* in the Caribbean and Gulf of Mexico. PCA was generated in adegenet (Jombart, 2008), a package in R designed for multivariate analysis of genetic markers data. For this graph only PC1 and PC2 are used and populations were color-classified into 3 groups based on STRUCTURE results (Table S3 in Appendix 1). Samples colored in Blue are full Caribbean populations (population assignment >0.7), the samples colored in Red are true Gulf of Mexico populations and have an assignment of >0.7 to cluster 2. The populations colored in Green are populations that share identity between the Red and Blue clusters and have an assignment between 0.3 and 0.7. The relative contributions of eigenvalues of each PC are presented in the inlayed graph.


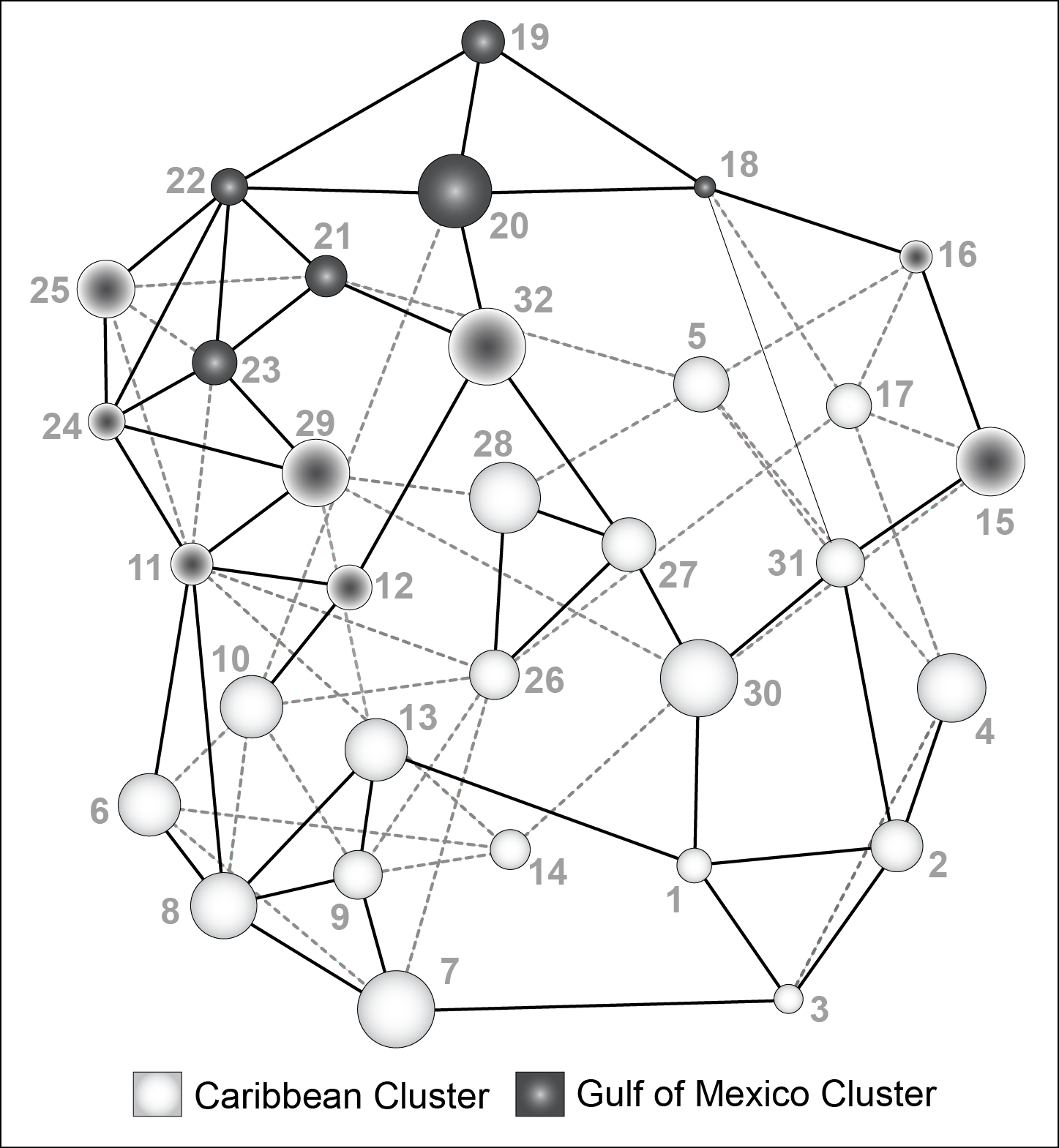


**Figure S5** Population Graph of *T. testudinum* in the Greater Caribbean and Gulf of Mexico based on the genetic diversity of 9 microsatellite loci created in GENETIC STUDIO b.131 (Dyer, 2009). Each sphere represents a sampled population (see Table 1 and Figure 1 respectively for names and location details of populations) and its size is proportional to the within-population genetic variance (Dyer & Nason, 2004). Node-shading represents one of the two identified biogeographic regions; Caribbean and Gulf of Mexico. Populations that had no strong affiliation to either region (See Table S3 in Appendix1) have intermediate shading. Lines (black and dotted) are retained edges that represent significant genetic relatedness, lengths correspond to the genetic distance relative to total genetic diversity. Both nodes and edges are rendered in a three-dimensional space, dotted lines are positioned deeper in the graph, solid lines are the superficial edges.

**

Figure S6** Connectivity graph of 32 populations of *Thalassia testudinum* in the Caribbean and the Gulf of Mexico. Relative migration between all 32 populations was calculated and plotted with the R package diveRsity (Keenan *et al.*, 2013) using the divMigrate function (Sundqvist *et al.*, 2013) based on *G*_ST_ (Table S14 in Appendix 2). The divMigrate function plots the relative asymmetric migration between populations, from microsatellite allele frequency data. A lower threshold of 0.14 was used to eliminate uninformative edges and edges were scaled by width and colour saturation above 0.4. Wider and darker edges represent the most connected sites of this study with the highest relative connectivity (1.0) between 23.US Craig Key and 24.US Arsenicker. The numbers within the nodes represent the populations as in Table 1.

**REFERENCES**

Dyer, R.J. (2009) GeneticStudio: a suite of programs for spatial analysis of genetic-marker data. *Molecular Ecology Resources*, **9**, 110-113.

Dyer, R.J. & Nason, J.D. (2004) Population Graph: the graph theoretic shape of genetic structure. *Molecular Ecology*, **13**, 1713-1727.

Evanno, G., Regnaut, S. & Goudet, J. (2005) Detecting the number of clusters of individuals using the software STRUCTURE: a simulation study. *Molecular Ecology*, **14**, 2611-2620.

Jombart, T. (2008) adegenet: a R package for the multivariate analysis of genetic markers. *Bioinformatics*, **24**, 1403-1405.

Keenan, K., McGinnity, P., Cross, T.F., Crozier, W.W. & Prodöhl, P.A. (2013) diveRsity: An R package for the estimation and exploration of population genetics parameters and their associated errors. *Methods in Ecology and Evolution*, **4**, 782-788.

Kopelman, N.M., Mayzel, J., Jakobsson, M., Rosenberg, N.A. & Mayrose, I. (2015) Clumpak: a program for identifying clustering modes and packaging population structure inferences across K. *Molecular Ecology Resources*, **15**, 1179-1191.

Pritchard, J.K. & Wen, W. (2003) Documentation for STRUCTURE software: Version 2.

Sundqvist, L., Zackrisson, M. & Kleinhans, D. (2013) Directional genetic differentiation and asymmetric migration. *ARXIV*, eprint arXiv:1304.0118.
